# κO-SrVIA Conopeptide, a Novel Inhibitor Peptide for Two Members of the Human EAG Potassium Channel Family

**DOI:** 10.3390/ijms241411513

**Published:** 2023-07-15

**Authors:** Luis Martínez-Hernández, Estuardo López-Vera, Manuel B. Aguilar, Ximena C. Rodriguez-Ruiz, Mónica A. Ortíz-Arellano

**Affiliations:** 1 Posgrado en Ciencias Biológicas, Instituto de Ciencias del Mar y Limnología, Universidad Nacional Autónoma de México, Ciudad de México 04510, Mexico; lamh14@gmail.com; 2 Laboratorio de Toxinología Marina, Unidad Académica de Ecología y Biodiversidad Acuática, Instituto de Ciencias del Mar y Limnología, Universidad Nacional Autónoma de México, Ciudad de México 04510, Mexico; himena_dark@hotmail.com; 3 Laboratorio de Neurofarmacología Marina, Departamento de Neurobiología Celular y Molecular, Instituto de Neurobiología, Universidad Nacional Autónoma de México, Juriquilla 76230, Mexico; maguilar@unam.mx; 4 Laboratorio de Malacología, Facultad de Ciencias del Mar, Universidad Autónoma de Sinaloa, Mazatlán 82000, Mexico; manabel@uas.edu.mx

**Keywords:** *Conus spurius*, conopeptides, conotoxins, EAG channels, Kv10.1 channel, Kv11.1 channel, Kv voltage-gated potassium channels

## Abstract

The first conotoxin affecting the voltage-gated potassium channels of the EAG family was identified and characterized from the venom of the vermivorous species *Conus spurius* from the Gulf of Mexico. This conopeptide, initially named Cs68 and later designated κO-SrVIA, is extremely hydrophobic and comprises 31 amino acid residues, including six Cysteines in the framework VI/VII, and a free C-terminus. It inhibits the currents mediated by two human EAG subtypes, Kv10.1 (IC_50_ = 1.88 ± 1.08 µM) and Kv11.1 (IC_50_ = 2.44 ± 1.06 µM), and also the human subtype Kv1.6 (IC_50_ = 3.6 ± 1.04 µM). Despite its clear effects on potassium channels, it shares a high sequence identity with δ-like-AtVIA and δ-TsVIA. Also, κO-SrVIA is the third conopeptide from the venom of *C. spurius* with effects on potassium channels, and the seventh conotoxin that blocks Kv1.6 channels.

## 1. Introduction

Marine snails belonging to the genus *Conus* are a group characterized mainly by the beauty of the color patterns on their shells and the great diversity of toxins contained in their venom apparatus, first detected by reports of poisoning in humans and the bioassays of the crude venom performed by Endean and Rudkin (1965) [1,2]. It is well known that these marine snails use the venom to capture their prey, worms, fishes, and other mollusks [3].

The venoms of *Conus* are generally composed of peptide molecules between 12 to 40 amino acid residues in length. These molecules are known as conopeptides and they have been documented to have remarkable affinity for a wide range of molecular targets such as voltage-gated ion channels (Nav^+^, Kv^+^, Cav^2+^), ligand-gated ion channels (nAChR, 5-HT_3_R, NMDAR), neurotransmitter transporters (for noradrenaline and norepinephrine) and G-protein-coupled receptors (for somatostatin) [4,5,6,7,8]. Of the more than 700 species that make up the genus, only thirteen conopeptides with activity on potassium channels from nine species have been characterized until now [9,10].

In mammals, the alpha subunits that form potassium channels are encoded by more than 90 genes [11]. In normal conditions, potassium channels mediate the electrical activity in those cells where they are expressed; for instance, they control the neuronal action potential (Kv1.1 and Kv1.2), the cardiac interval (Kv7.1 and Kv11.1) [12], and high-frequency stimulus trains in some brain regions (Kv10.1) [13]; indeed, they stop hyperexcitability upon traumatic nerve injury (Kv1.6) [14]. Under abnormal conditions, these subunits are related to diseases in humans called channelopathies. These channelopathies can be classified into two types: i) gain/loss of function and ii) ectopic expression. The first refers to mutations in the coding genes, which results in improper functioning of the channel; for example, a mutation in the KCNH2 gene that encodes the cardiac Kv11.1 channel induces an arrhythmia known as long QT syndrome [15]. The second refers to an expression where the channel is not normally expressed; an example of this is the KCNH1 gene that encodes the Kv10.1 channel, whose overexpression has been reported in a wide variety of tissues that present tumor growth, which is why it is considered a tumor marker [16].

Given the roles of the Kv10.1 channel, in the present work, we aimed to identify conotoxins affecting this subtype. Electrophysiological assays on *Xenopus laevis* oocytes allowed us to discover a new conopeptide called Cs68 (κO-SrVIA), which exerts inhibitory activity upon Kv10.1, Kv11.1, and Kv1.6 potassium channels, with a slightly higher affinity for the Kv10.1 channel.

## 2. Results

### 2.1. Conus spurius Venom Fractionation and Purification of Fraction Cs68 Subsection

**Figure 1:**
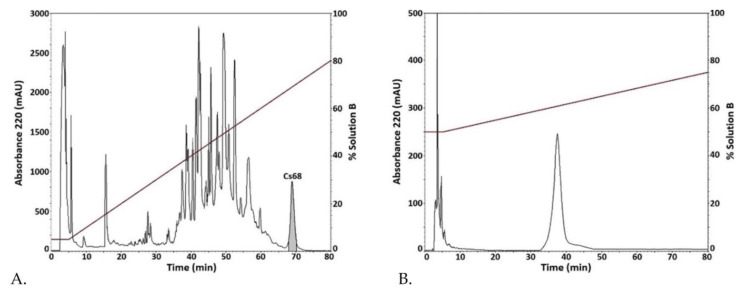
Purification of fraction Cs68 by RP-HPLC. (**A**) Chromatogram profile of the fractionation of the crude venom from *C. spurius*. After 5 min at 5% Solution B, a linear gradient of 5 to 80% Solution B in 75 min was applied at flow rate of 1 mL/min. (**B**) The fraction highlighted in (**A**) was subfractionated using an isocratic step of 50% Solution B for 5 min, followed by an elution gradient of 50 to 75% Solution B in 75 min at a flow rate of 1 mL/min.

In order to identify any component with inhibitory activity on the Kv10.1 potassium channel, electrophysiological evaluation of different fractions of *C. spurius* venom obtained by RP-HPLC was performed; the 24 apparently most abundant fractions were assayed at 0.041 µg/µL. Fraction Cs68, which gets its name because it elutes at minute 68 during fractionation, was the only fraction that had inhibitory activity on the Kv10.1 channel (Figure 1A). One additional step of purification by RP-HPLC was necessary to obtain a pure venom component (peptide Cs68) (Figure 1B).

### 2.2. Molecular Mass and Amino Acid Sequence Analysis

**Figure 2:**
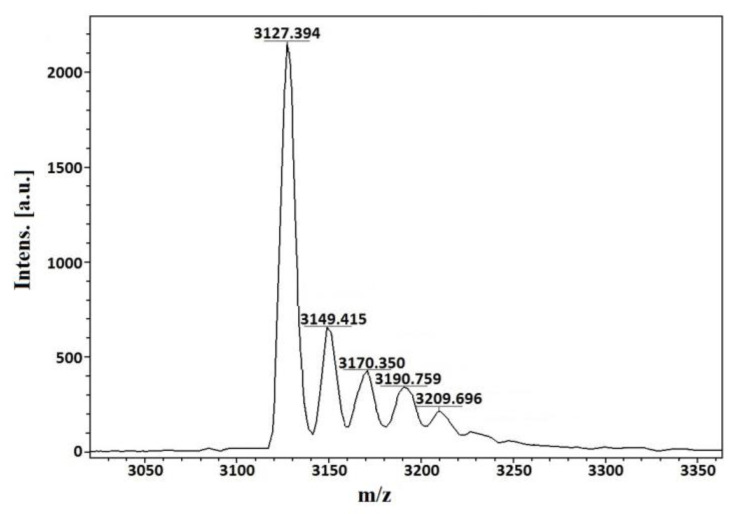
MALDI-TOF spectrum of purified fraction Cs68. The average *m*/*z* (*z* = +1) signal at 3127.394 corresponds to an average mass of 3126.386 Da.

The mass spectrum of peptide Cs68 displays a clean major *m*/*z* (*z* = +1) signal at 3127.394 (Figure 2), that corresponds to an average mass of 3126.386 Da. The signal at 3149.415 differs from the previous one by 22.02 *m*/*z* units and corresponds to a sodium adduct.

**Figure 3:**
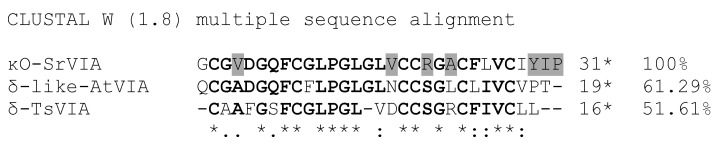
Amino acid sequence of peptide Cs68 (renamed as κO-SrVIA; see Section 3) and comparison with δ-like-AtVIA and δ-TsVIA. Asterisks indicate identical residues among all the sequences; colons denote conserved substitutions; dots indicate semiconserved substitutions. Identical residues in two or three sequences are highlighted in boldface, whereas residues in κO-SrVIA that have clearly different physicochemical properties with respect to the δ-conotoxins are highlighted in gray background. The figures followed by an asterisk are the numbers of identical residues compared to κO-SrVIA, whereas the numerals followed by a percent sign are the percents of identical residues compared to κO-SrVIA.

Thirty-two cycles of automated Edman degradations were performed and a sequence of 31 amino acids was obtained. Position 2, 9, 18, 19, 23, and 27 were blank cycles, which we assume correspond to cysteine residues. The theoretical average mass of the sequence, considering three disulfide bonds, is 3128.78 Da if a free C-terminus is presumed, and 3127.80 Da if a C-terminal amide is supposed. Due to the difference between the experimental and each theoretical values, we searched for the sequence of peptide Cs68 in the transcriptome of the venom gland of *C. spurius*, recently published [17]; the sequence is identical to the mature region predicted from Assembly/Isolate DN54709c1g1i1151/Sr6.O.04 by the ConoPrec tool of the ConoServer database [18]; in this precursor the C-terminus is a proline residue, which rules out the possibility of amidation (Figure 3).

### 2.3. Similarity Search

BLASTp similarity search for the Cs68 sequence gave six hits with an E value better than 4 × 10^−8^, including two for conotoxins for which a target has been identified: δ-like-AtVIA from the worm hunter snail *C. ateralbus* (two hits with percent identity of 70.37% and query coverage of 87%) and δ-TsVIA (one hit with percent identity of 59.26% and query coverage of 87%) (Figure 3). 

### 2.4. Electrophysiological Evaluation of Cs68 on Voltage-Gated Potassium Channels

**Figure 4:**
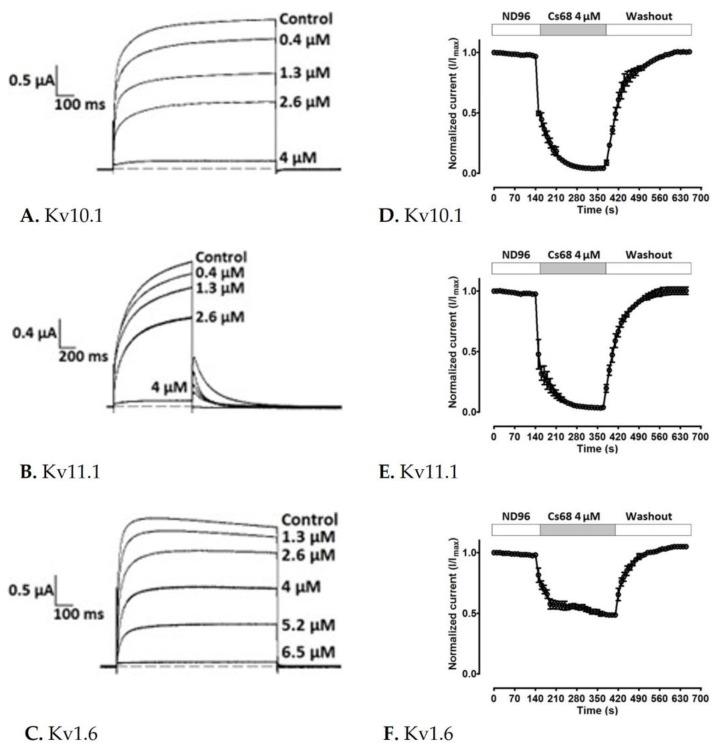
Inhibition of K^+^ channels by Cs68. Representative recording showing control currents and in presence of different concentrations of Cs68 A on Kv10.1 (**A**), Kv11.1 (**B**), and Kv1.6 (**C**). Two electrode voltage clamp recordings were made from channels expressed in oocytes. Currents were evoked by voltage steps to +10 mV from a holding potential of −80 mV applied every 10 s. Time course of inhibition by Cs68 at 4 µM (static flow) from the normalized current (I/Imax) on Kv10.1 (**D**), Kv11.1 (**E**), and Kv1.6 (**F**).

**Figure 5:**
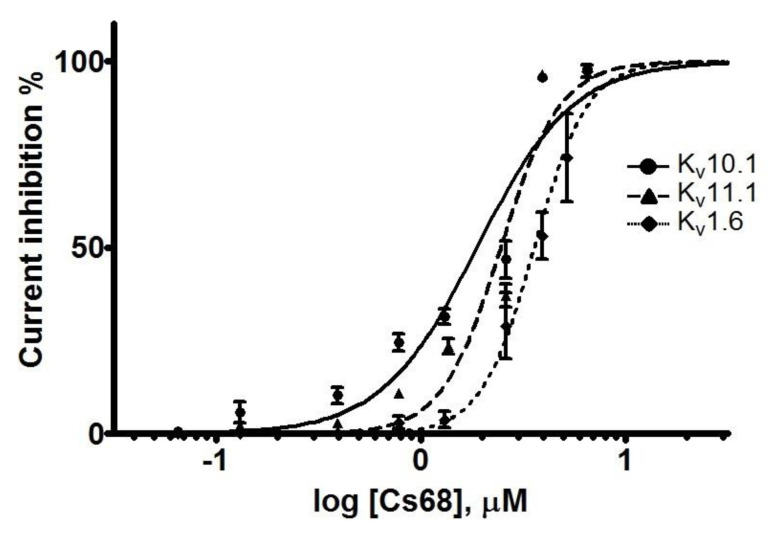
Concentration-response curves of Cs68 over different Kv channel subtypes. Cs68 inhibits all subtypes Kv10.1 (IC_50_ = 1.88 ± 1.08 µM, Hill coefficient 1.86 ± 0.25), Kv11.1 (IC_50_ = 2.44 ± 1.06 µM, Hill coefficient 2.85 ± 0.46), and Kv1.6 (IC_50_ = 3.6 ± 1.04 µM, Hill coefficient 3.35 ± 0.44). *n* = 3 for each concentration; error bars = standard error of the mean (SEM).

Fraction Cs68 was initially evaluated by TEVC in *X*. *laevis* oocytes expressing the voltage-dependent K^+^ channel Kv10.1. Cs68 showed 100% inhibitory activity on the currents at 4 µM. Hence, we evaluated Cs68 on two more subtypes of K^+^ channels where other compounds with effects on Kv10.1 also have shown activity (Kv11.1 and Kv1.6) [9,19]. We found that at the low concentration of 0.4 µM the inhibitory activity was 10% on Kv10.1 and Kv11.1 (Figure 4A,B), but no effect on Kv1.6 was observed. However, at 4 µM the inhibition was less than 50% on Kv1.6 but 100% on Kv11.1 (Figure 4C). Figure 4D–F show the time course of inhibition of the 3 subtypes of K^+^ channels by Cs68. Then, concentration-response curves were generated for Kv10.1, Kv11.1, and Kv1.6 channels (Figure 5). Peptide Cs68 presented a slightly higher affinity for Kv10.1 (IC_50_ 1.88 ± 1.08 µM) with respect to Kv11.1 (IC_50_ 2.44 ± 1.06 µM) and Kv1.6 (IC_50_ 3.6 ± 1.04 µM).

## 3. Discussion

In this work, we identified a new peptide, Cs68, from the venom of the vermivorous species *Conus spurius*; the amino acid sequence of the peptide consists of 31 amino acids with three disulfide bridges and a free C-terminus. Since Cs68 has activity on potassium channels, and, in accordance with Zamora-Bustillos et al. (2021), this conopeptide belongs to the superfamily O1 and presents the cysteine pattern VI/VII, we decided to name it κO-SrVIA following the proposed nomenclature for conotoxins [17]. The disulfide connectivity of this new conopeptide remains unknown; however, we assume that it could be similar to that supposed for δ-like-AtVIA (I–IV, II–V, III–VI) with which it shares 70.3% of its amino acid sequence [20].

Electrophysiological tests with κO-SrVIA revealed that it is able to inhibit the currents elicited by three subtypes of voltage-gated potassium channels, Kv1.6, Kv10.1, and Kv11.1, at different concentrations. To date (June 2023) there are only thirteen conopeptides with activities on potassium channels and six of them have effects on Kv1.6: κJ-PlXIVA, CPY-Fe1, CPY-Pl1, κM-RIIIJ, κ-SrXIA (peptide sr11a), and CNF-Sr3 [21,22,23,24,25]. Therefore, Kv1.6 is the most common potassium channel target subtype for conopeptides. In this sense, κO-SrVIA would be the seventh conopeptide reported with activity on this subtype, and the third one isolated from the venom of *C. spurius* that blocks it, but, nevertheless, the first one with inhibitory activity on subtypes Kv10.1 and Kv11.1.

The identification of a third component in the venom of *C. spurius* with inhibitory activity on potassium channels suggests ecological importance. In 1996, Terlau and collaborators proposed that the presence of more than one potassium channel blocker in the venom of a *Conus* species may be due to the fact that they are used during prey capture, specifically in the rapid immobilization component [26]. It is important to highlight that, despite the fact that this strategy was proposed for snails that feed on fish, it could be shared by worm hunting snails as are *C. spurius* (toxins κ-SrXIA, CNF-Sr3, and κO-SrVIA) and *C. planorbis* (toxins kJ-PIXIVA and CPY-Pl1).

The study of conopeptides with activity in potassium channels has gained interest since the characterization of κO-PVIIA and its selectivity for the *Shaker* subtype with respect to other subtypes (Kv1.1 and Kv1.4) [26]. Taking this into account, the identification of new conopeptides that could serve as molecular tools to study the functionality of potassium channels has been the subject of study, especially when specific subtypes are involved in the development of channelopathies such as the cases of Kv10.1 and Kv11.1, implicated in the advancement of childhood epilepsy, Temple-Baraitser syndrome [27], Zimmermann-Laband syndrome [28], tumor growth [16], and long QT syndrome [15].

Considering the above, κO-SrVIA (Cs68) could represent a promising molecule to study the Kv10.1 and Kv11.1 channels. However, its usefulness may be limited by the similar affinity it presents for these two subtypes of potassium channels. A problem that has been encountered when looking for molecules with specific activities on subtype Kv10.1 is that they also inhibit the Kv11.1 [19]. Nevertheless, to solve this problem a strategy that includes the design of analogs based on natural conopeptides with interesting properties, such as κ-RIIIK and κ-RIIIJ from *C. radiatus,* can be applied; these analogs are designed to determine which amino acids are responsible for the activity and thus promote selectivity for subtypes of potassium channels [23].

The importance of studying molecules contained in the venom of different animals that interact with Kv10.1 potassium channels rose after the finding by Dr. Pardo and collaborators in 1999 that inhibition of Kv10.1 channels causes reduced cell proliferation in different cancer cell lines [29]. At the moment, four toxins have been isolated that target Kv10.1: κ-hefutoxin (*Heterometrus fulvipes*), APETx4 (*Anthopleura elegantissima*), Aa1a (*Avicularia aurantiaca*), and Ap1a (*Avicularia purpurea*). 

κ-hefutoxin has an IC_50_ of 26 µM on Kv10.1 [30] but inhibits Kv1.3 [31]; the IC_50_ of APETx4 for Kv10.1 is 1.01 µM and at a concentration slightly higher of 1.6 µM reduces the currents amplitude by 66% on Kv1.4 and by 50% on Nav1.4 channels [32]; Aa1A and Ap1A are the toxins that have higher affinities for Kv10.1 as their IC_50_ are 637 nM and 236 nM, respectively, with no activity on Kv11.1 until the concentration increases to 8 µM [33]. However, only Aa1a presents 100% of inhibition for Kv10.1 with a concentration of 20 µM; the same concentration for Ap1a produces less than 80% of inhibition [33]; a concentration of 80 µM of κ-hefutoxin reaches 90% of inhibition and APETx4 at 3 µM causes 88% of inhibition [32]. It is worth mentioning that, for Kv10.1, Ap1a and Aa1A have a Hill coefficient of 1.32 ± 0.13 and 1.03 ± 0.09 [33], respectively, while APETx4 has a coefficient of 3.8 ± 0.03 [32]; these values are close to those obtained with κO-SrVIA for the three subtypes of K^+^ channels. A Hill coefficient of ~1 normally indicates a single binding site while a higher Hill coefficient (~4 for APETx4) suggests possible cooperativity. Cooperativity might occur in the interaction between κO-SrVIA and the Kv10.1, Kv11.1, and Kv1.6 channels, but more experiments would be necessary to confirm this possibility.

In this sense, this work could contribute to the study of Kv10.1 due to the characteristics that κO-SrVIA has: it reaches 100% inhibition with a concentration of 4 µM (Figure 5), which is lower than the concentrations of the previously mentioned toxins; it has an IC_50_ similar to APETx4 (1.88 µM vs. 1.01 µM, respectively), and the length (31 amino acids) is similar to Aa1A and Ap1a. In other words, κO-SrVIA gathers all the characteristics of these toxins in one.

## 4. Materials and Methods

### 4.1. Isolation of Crude Venom Extract from Conus spurius

Six venom ducts from *Conus spurius* (stored at −70 °C) were homogenized in 10 mL of 40% (*v*/*v*) aqueous acetonitrile (ACN) and 2% (*v*/*v*) trifluoracetic acid (TFA) with a tissue homogenizer (BioSpec 985370 Tissue-Tearor; Bartlesville, OK, USA) at 4 °C. The resulting mix was centrifuged at 14,000× *g* for 15 min. The supernatant was decanted and the pellet was kept as the fraction rich in peptides. The protein content was quantified with a Nanodrop spectrophotometer at 280 nm (Nanodrop 2000, Thermo Fisher, Waltham, MA, USA).

### 4.2. Crude Venom Extract Fractionation by RP-HPLC

Aliquots of 7.5 mg of crude venom were dissolved with 1 mL of Solution A (aqueous solution with 0.1% (*v*/*v*) TFA) before fractionation by Reversed-Phase High Performance Liquid Chromatography (RP-HPLC) (Agilent Technologies, Infinity 1260 Series HPLC System; Santa Clara, CA, USA) using a Vydac Reverse-Phase Peptide and Protein C18 column (218TP54, 5 µm particle size, 4.6 mm × 250 mm) attached to a Vydac Reverse-Phase Peptide and Protein C18 Precolumn (218GK54, 5 µm particle size, 4.6 mm × 10 mm).

Fractions of the crude venom extract were eluted with an isocratic step of 5% Solution B (90% ACN in water containing 0.085% (*v*/*v*) TFA) for 5 min, followed by a linear gradient from 5% to 80% (*v*/*v*) of Solution B in 75 min, at a flow rate of 1 mL/min. Further purification of a selected fraction (Cs68) was performed by RP-HPLC using an isocratic step at 17% of Solution B for 5 min, followed by a linear gradient from 50% to 75% (*v*/*v*) of Solution B, over 75 min at a flow rate of 1 mL/min. The elution profiles were monitored at a wavelength of 220 nm. Each signal was collected manually.

### 4.3. Amino Acid Sequencing and Mass Spectrometry Analysis

The molecular mass of the Cs68 fraction was analyzed by MALDI-TOF with an α-cyano-4-hydroxycinnamic acid matrix at the facilities of the Chemistry Institute, UNAM.

To determine the amino acid sequence, one nmol of the peptide was analyzed by automated Edman degradation using a PPSQ-31A protein sequencer (Shimadzu Scientific Instruments; Tokyo, Japan) by Dr. Fernando Zamudio at the Biotechnology Institute, UNAM.

Theoretical masses were calculated using the Peptide Mass Calculator tool of the Ion Source-Mass Spectrometry Educational Resource website (https://www.ionsource.com/).

### 4.4. Preparation of Vectors Encoding Potassium Channels

cDNA of the clones for human Kv10.1, Kv11.1 and Kv1.6 channels in vector pSGEM, resistant to ampicillin, were linearized with SpHI, Sfil, and EcoRI, respectively. Then, the products were purified using a QIAquick PCR purification Kit (QIAGEN; Redwood City, CA, USA). cDNAs were transcribed in vitro with T7 polymerase (mMessage mMachine Kit; Ambion Inc., Austin, TX, USA). cRNA was purified using a Qiagen RNeasy kit (QIAGEN; Redwood City, CA, USA). 

### 4.5. Expression of Voltage-Gated Potassium Channels

For heterologous expression, stage V-VI *Xenopus laevis* oocytes were isolated by microsurgery. Sexually mature *Xenopus leavis* female frogs were kept and handled according to the institutional bioethics committee requirements. The frogs were anesthetized by a 30 min submersion in 2% tricaine methane sulfonate (MS-222; Merck KGaA, Darmstadt, Alemania) before performing microsurgery. Isolated oocytes were defolliculated with 0.75 mg/mL collagenase A (Roche; San Jose, CA, USA; Cat. # 10103586001) and kept in ND96 extracellular solution (96.0 mM NaCl, 2.0 mM KCl, 1.0 mM MgCl_2_·6 H_2_O, 1.8 mM CaCl_2_·2 H_2_O, 5 mM HEPES, pH 7.3) supplemented with penicillin/streptomycin (100 U/100 µg)/mL and 100 µg/mL gentamicin at 15 °C.

Thirty nanograms of Kv1.6, 9.8 ng of Kv10.1, and 18 ng of Kv11.1 of cRNA were injected into each defolliculated oocyte using a micro-injector Nanoliter 2000 (World Precision Instruments; Sarasota, FL, USA).

### 4.6. Electrophysiological Recording 

Whole-cell currents were recorded under two-electrode voltage clamp (TEVC) using an OC-725C clamp amplifier (Warner Instruments; Holliston, MA, USA) between 24 to 72 h after injections of the genetic material. Data acquisitions were done with the program *LabView* (National Instruments, Chihuahua, Mexico). The intracellular electrodes were filled with 3 M KCl and selected for resistance between 0.6–1 MΩ. Potassium currents were generated by depolarizations to +10 mV for 1 s every 10 s from a membrane potential held at −80 mV. The bath solution was ND96, consisting of 96 mM NaCl, 2.0 mM KCl, 1.8 mM CaCl2, 1.0 mM MgCl2, and 5 mM HEPES (pH 7.2) with pen/strep (100 U/100 µg)/mL and 100 µg/mL of gentamicin. To assay toxin Cs68, we stopped the flow and directly applied it (diluted in ND96, 3 µL) to the bath chamber (30 µL); once maximum inhibition was reached at a given concentration, the flow for toxin washout was restarted.

All electrophysiological measurements were performed at room temperature (≈22 °C). The duration of records was 1500 ms for Kv1.6 and Kv10.1, and 3000 ms for Kv11.1.

Measurements are reported as the mean ± standard error of the mean (SEM) of three independent experiments. The current inhibition % was calculated from the current amplitude values obtained with the ND96 extracellular solution (I_Control_) and those of each of the experimental conditions (I_[Cs68]_); therefore, Current inhibition % = [1 − (I_[Cs68]_/I_Control_)] × 100. Current plots were made with SigmaPlot version 10 (Systat Software, Inc., San Jose, CA, USA). The construction of the concentration–response curve and the determination of the mean inhibitory concentration (IC_50_) were obtained from the data of the current inhibition % as a function of the concentration (µM) of Cs68 expressed in logarithm (log[Cs68]) using GraphPad Prism version 9.5.1 (GraphPad Software, San Diego, CA, USA). Concentration–response curves were fit to the equation: Current inhibition % = 100/{1 + (log[Cs68]/IC_50_)*^n^*^H^}, where *n*H is the Hill coefficient.

### 4.7. Similarity Search and Sequence Comparison

The standard protein–protein Basic Local Alignment Search Tool (protein–protein BLAST, NCBI, 13 February 2023) was employed to search the non-redundant protein sequences database with organism *Conus* (taxid: 6490) using the default settings.

Sequence alignments were generated using Clustal W version 1.8 [34] at the PRABI-Gerland Rhone-Alpes Bioinformatic Pole Gerland Site, Institute of Biology and Protein Chemistry at https://npsa-prabi.ibcp.fr/cgi-bin/npsa_automat.pl?page=npsa_clustalw.html, with the default parameters [35].

## 5. Conclusions

Considering the pros and cons of the toxin characterized in this study compared with the other toxins mentioned above, we can say that κO-SrVIA is a promising molecule for the study of potassium channels and can act as a template for designing analogs that permit us to understand the mechanism underlying the function of subtypes Kv10.1 and Kv11.1.

## 6. Patents

Patent pending. 

## Data Availability

Not applicable.
